# Toward an Optimal Diet for Health in Asia: The Asian–Mediterranean Dietary Pattern

**DOI:** 10.1016/j.advnut.2026.100727

**Published:** 2026-08-27

**Authors:** Jiaxi Yang, Chuwen Ling, Ying Ting Er, Yap Seng Chong, Frank B Hu, Laurent Hochet, Miguel A Martínez-González, Christiani Jeyakumar Henry, Cuilin Zhang

**Affiliations:** 1 Global Center for Asian Women’s Health, Yong Loo Lin School of Medicine, National University of Singapore, Singapore; 2 Department of Obstetrics and Gynaecology, Yong Loo Lin School of Medicine, National University of Singapore, Singapore; 3 Bia-Echo Asia Center for Reproductive Longevity and Equality, Yong Loo Lin School of Medicine, National University of Singapore, Singapore; 4 Department of Nutrition, Harvard T.H. Chan School of Public Health, Boston, MA, United States; 5 Department of Medicine, The Channing Division for Network Medicine, Brigham and Women's Hospital and Harvard Medical School, Boston, MA, United States; 6 Sodexo Singapore, Singapore; 7 Navarra Institute for Health Research (IdiSNA), University of Navarra, Pamplona, Spain; 8 Department of Preventive Medicine and Public Health, University of Navarra, Pamplona, Spain; 9 Biomedical Research Network Centre for Pathophysiology of Obesity and Nutrition (CIBEROBN), Carlos III Health Institute, Madrid, Spain

**Keywords:** Mediterranean diet, Asian diets, Asian–Mediterranean diet, dietary pattern, nutrition transition, noncommunicable diseases, healthy aging

## Abstract

With rapid population aging and lifestyle transitions, Asian countries are facing a growing burden of diet-related noncommunicable diseases (NCDs). Optimal nutrition is central to preventing NCDs and promoting healthy aging. Traditional Asian diets have many strengths, including abundant vegetables, plant-based proteins, functional foods, and nutrient-preserving cooking methods such as steaming and stewing. However, they also have limitations, including widespread use of refined carbohydrates and high sodium intake. In recent decades, the nutrition transition toward energy-dense, highly processed diets has further exacerbated these existing challenges and contributed to declining dietary quality. Despite efforts across Asia to establish healthy dietary frameworks, the region’s diverse food cultures and limited research make it challenging to conceptualize a unified, culturally appropriate, and evidence-based dietary pattern. The Mediterranean diet has gained global recognition for its health benefits. However, its direct application in Asia is limited by cultural, culinary, and food system differences. In this Perspective article, the Asian–Mediterranean diet (AMED) is proposed as a culturally adapted framework that preserves the evidence-based principles of the Mediterranean diet while integrating healthy Asian foods and culinary practices. It emphasizes plant-based proteins such as soy products, healthy plant oils like canola and sesame oils, whole grains including brown rice and millet, abundant seasonal fruits and vegetables, seafood and poultry, and traditional functional foods such as fermented foods and teas. Nutrient-preserving cooking methods, together with moderate use of condiments, are encouraged. A 20-wk randomized controlled trial in Singapore is underway to evaluate the feasibility, acceptability, adherence, and health effects of AMED. As evidence accumulates, AMED has the potential to provide a scalable strategy that bridges Mediterranean evidence with Asian food cultures, promoting healthier and longer lives across Asian populations.

**Statement of significance**

Dietary strategies are among the most cost-effective approaches to reducing the burden of noncommunicable diseases (NCDs) and promoting population longevity. Although the concept of an Asian-adapted Mediterranean diet has previously been discussed, this Perspective article goes beyond existing literature by providing one of the first comprehensive, evidence-based frameworks that synthesizes evidence on Mediterranean dietary principles, traditional Asian dietary patterns, locally available foods, and cooking methods, underscoring why such an approach is needed and how it can be translated into a culturally appropriate dietary pattern for diverse Asian populations. By integrating evidence synthesis with practical implementation and evaluation, this Perspective provides a roadmap for future research, policy development, and large-scale adoption of the Asian–Mediterranean diet across Asia.

## Introduction

Optimal nutrition is essential for health and well-being. The WHO emphasizes that maintaining a balanced, healthy diet across the life course protects against malnutrition and noncommunicable diseases (NCDs), such as type 2 diabetes (T2D), cardiovascular disease (CVD), stroke, and cancer [1]. Unhealthy, low-quality diets, by contrast, are among the leading global risk factors for poor health. Accordingly, numerous national and international health bodies have identified healthy dietary patterns as powerful strategies for chronic disease prevention and the promotion of healthy aging [1,2].

In parallel with increases in life expectancy, the concept of “health span,” defined as the number of years an individual is free from major illness or disability, has gained growing attention [3]. Global life expectancy rose from 66.8 y in 2000 to 73.1 y in 2019, but the population has aged, and the share of deaths attributable to NCDs also increased from 61% to 74% during the same period [4]. Asia has been at the center of this shift: despite regional variations, overall economic growth and social transformations have markedly reduced communicable disease mortality, leading to accelerated population aging and increased NCD risk in the region [5].

Asia possesses vast geographical, socioeconomic, biological, and cultural diversity, reflected in a wide range of traditional Asian dietary patterns. Despite this diversity, many share common features, including abundant vegetables [6], plant-based proteins such as tofu, legumes, and nuts [7], whole grains such as millet and barley [6], and fermented foods [8]. However, traditional Asian diets also have limitations, including reliance on refined carbohydrate staples, high sodium intake, and culinary practices associated with adverse health outcomes. In recent decades, many Asian countries have undergone a nutrition transition, with traditional diets increasingly displaced by energy-dense, highly processed diets that are poor in nutrients but high in animal products, refined carbohydrates, sugars, sodium, additives, and unhealthy fats [9]. Together, these dietary shifts and constraints contributed to declining dietary quality and the growing burden of NCDs across the region. As such, there is an urgent need for effective, culturally sensitive strategies to address both traditional and emerging dietary challenges while preserving the strengths of traditional Asian diets.

The concept of a healthy dietary pattern is globally relevant but can vary across food cultures and agricultural systems. The Mediterranean diet, one of the most extensively studied dietary patterns, emphasizes reductions in red and processed meats, sweets, ultraprocessed foods, and saturated fat, but abundant unprocessed or minimally processed plant-based foods, whole grains, legumes, nuts, olive oil, fish, and moderate consumption of poultry [10]. It is highly palatable and has consistently shown benefits for aging and prevention of NCDs in observational studies and randomized controlled trials (RCTs) primarily from Europe and North America, suggesting its potential for broader health promotion [11]. United Nations Educational, Scientific and Cultural Organization has officially recognized the Mediterranean diet as an Intangible Cultural Heritage of Humanity, highlighting its value as a set of traditional practices, knowledge, skills, and social customs that have been passed down across generations with scientifically recognized health benefits, particularly for the prevention of cardiometabolic diseases [11,12]. Its direct application in Asian populations—though likely hindered due to differences in diet culture, culinary preferences, and food availability—could represent an attractive option for the region [13,14]. The concept of an Asian-adapted Mediterranean diet has been proposed previously [15], and recent reviews discussed potential Asian food choices that align with Mediterranean dietary principles [15,16]. However, a comprehensive framework that systematically translates these principles into a culturally appropriate and practical dietary pattern by integrating the current evidence on healthy dietary patterns, regionally available foods, traditional cooking methods, culinary practices, and implementation considerations has yet to be established.

## Mediterranean Diet as a Benchmark for Healthy Diets

Over the years, several diets have drawn global attention for their potential for optimizing health and reducing NCD risks. The Mediterranean diet stands out as one of the most researched and widely popularized diets in Western countries. Because Ancel Keys proposed the Mediterranean diet concept [17], numerous studies have demonstrated that the Mediterranean diet has significant health benefits in preventing several NCDs.

Robust evidence from large cohort studies and RCTs primarily conducted in Europe and North America has established the Mediterranean diet as a benchmark for healthy eating. Prospective cohort studies consistently show that greater adherence to the Mediterranean diet is associated with reduced risks of all-cause mortality in the general population, including cardiovascular, cancer-related, and dementia-related deaths [18,19]. Protective associations have also been reported for the incidence of major cardiometabolic diseases, such as CVD [20] and T2D [21], as well as for diet-related cancers, particularly colorectal cancer [22] and postmenopausal breast cancer [23]. Additional benefits include reduced frailty [24], improved cognitive health, and lower Alzheimer’s disease risk in older adults [25]. Causal evidence is further provided by several large-scale RCTs. The “Prevención con Dieta Mediterranean” (PREDIMED) trial, a primary prevention trial of CVD conducted in Spain, randomly assigned 7447 individuals at high cardiovascular disease risk to 1 of 3 diets: a Mediterranean diet supplemented with extra-virgin olive oil (EVOO), a Mediterranean diet supplemented with mixed nuts, or a control diet focused on overall fat reduction. After a median follow-up of 4.8 y, the EVOO and nut groups experienced 31% and 28% relative reductions in major cardiovascular events, respectively, compared with the control group receiving repeated advice on a low-fat diet [26]. Secondary analyses from PREDIMED demonstrated reduced risks of T2D, subclinical CVD, and breast cancer, as well as improved cognitive health [[27], [28], [29]]. Other RCTs in Europe among high-risk populations also reported similar findings [30]. Beyond these health outcomes, a 2022 systematic review highlighted the benefits of the Mediterranean diet for women’s reproductive health over the life course, including enhanced fertility, pregnancy outcomes, and menopausal transition [31]. Emerging evidence also suggests intergenerational benefits: greater maternal adherence to the Mediterranean diet during pregnancy has been associated with favorable outcomes in offspring [32].

The Mediterranean diet is a predominantly plant-based dietary pattern that emphasizes minimally processed foods, consistent with many evidence-based dietary models [18]. A distinguishing feature of the Mediterranean diet is its emphasis on healthy plant-based fats, particularly olive oil and nuts. This feature challenges the early dietary guidelines on “reducing total fat” common in the United States during the 1980s [11]. Accumulating data now support the health benefits of vegetable oils high in unsaturated fatty acids (e.g., olive oil, rapeseed oil, and canola oil) and nuts, including improved body composition and lipid profiles and reduced risks of cardiovascular, metabolic, and cognitive diseases [33,34].

Mechanistically, the Mediterranean diet is low in saturated fats and rich in unsaturated fats, dietary fiber, bioactive compounds, and essential micronutrients. These features are hypothesized to enhance glycemic control and lipid metabolism, reduce inflammation and oxidative stress, and modulate gut microbiome composition [35], central to mechanisms implicated in major NCDs and the biological process of aging. Supporting this, adherence to the Mediterranean diet has been linked to longer telomere length, a biomarker of cellular aging [36].

In recent years, research on Mediterranean diet-based patterns has increased across Asia [37,38]. However, the “Mediterranean” diets examined in these studies are largely heterogeneous, reflecting different applications of the diet to Asian populations. Given the robust evidence supporting the health benefits of the Mediterranean diet, alongside Asia’s longstanding traditions of healthful eating patterns and diverse food sources, there is a timely opportunity to develop a culturally adapted dietary model that bridges and leverages these strengths. This motivates the development of the Asian–Mediterranean diet (AMED), which maintains the principles of the Mediterranean diet while integrating healthy elements of Asian cuisines. Rather than posing a barrier, Asia’s culinary and agricultural diversity can facilitate the application of the Mediterranean diet principles by offering greater flexibility and flavor. Importantly, preserving and incorporating healthy elements of traditional Asian diets is essential for maximizing AMED’s health benefits and ensuring cultural relevance [11].

## Healthy Traditional Asian Diets

Some dietary patterns or compositions in Asian cultures are known to maintain overall health. Understanding these elements can provide direction for building sustainable and healthy dietary models that align with local traditions. We will discuss them below from 3 levels: dietary patterns, food composition, and cooking methods.

Many traditional Asian diets often exhibit a well-structured composition, portion sizes, and food diversity [39,40]. For example, traditional Japanese diets often involve eating small portions of each food while maintaining high nutritional density and balance [39]. They are characterized by a high intake of isoflavone-rich soy products, fish rich in omega-3 (*n*–3) fatty acids, green tea, and relatively low intakes of red meat and saturated fat [39]. A diverse diet contributes to NCD prevention [41] and healthier aging, including cognition, frailty, and mental and sleep health, as suggested in prior systematic reviews [42]. The Japan Public Health Center–based Prospective Study found that these dietary characteristics of traditional Japanese diets were associated with lower risks of cancer, CVD, and T2D [43].

A notable example of traditional Japanese diets is Okinawa’s dietary heritage, commonly known as the Okinawa diet [14]. It features high intakes of root vegetables (primarily sweet potatoes), green and yellow vegetables, soy-based foods, marine products, lean meats, fruits, medicinal toppings and spices, and tea [44]. Compared with other regional Japanese diets, the Okinawa diet is lower in salt intake, as reflected by lower urinary sodium excretion levels among Okinawan residents than the national mean [45]. A retrospective 6-decade cohort study of local older residents found that the diet was associated with lower mortality from age-related diseases and increased life expectancy [46].

Outside Japan, because of different geography and dietary cultures, China also exhibits a few regional healthy eating patterns. The Jiangnan diet, primarily popular in Southeast China, emphasizes a diet rich in seasonal vegetables and fruits, freshwater fish and shrimp, legumes, moderate consumption of whole-grain rice (usually mixed with white rice), vegetable oils (mainly rapeseed oil), and red meat, together with relatively low intake of salt and millet wine. Steaming, boiling, and stir-frying are the main cooking methods [40]. A 6-mo RCT showed that the isocaloric-restricted Jiangnan diet achieved comparable weight loss and improved glucose homeostasis to the isocaloric-restricted Mediterranean diet among Chinese individuals at metabolic risk [37].

Traditional diets in South and Southeast Asia reflect the local people’s healthy dietary patterns to varying degrees. The traditional Indian diets include a rich mix of cereals, as well as pulses, oilseeds, fruits, vegetables, herbs, spices, and dairy products [47]. Typical Vietnamese diets include rice, soup, marine products, pork, vegetables, and tropical fruits [48]. When prepared traditionally, these diets often contain modest intakes of energy-dense animal foods and fats, generally in line with modern principles of satiety, nutrient balance, and preventive nutrition.

Collectively, these regional diets share key features with the Mediterranean diet—palatability; an emphasis on plant-based foods, oils replacing butter, cream, or margarine, and fish replacing meat; minimally processed foods; and the use of local fresh ingredients and traditional cooking styles.

Some commonly consumed Asian foods have been associated with health benefits. For example, fermented foods such as kimchi, natto, miso, and tempeh may offer probiotic effects, bioactive peptides, biogenic amines, and phenolic compound conversion, while reducing antinutrients [49]. Among these fermented foods, natto [50] and kimchi [51] have been evaluated in relation to health biomarkers in RCTs. No direct causal evidence has been shown for miso or tempeh, although a limited number of in vitro and observational studies have reported some promising findings [52,53]. In addition to fermented foods, green tea, a traditional beverage in East Asia, is rich in antioxidant catechins and has been linked to delayed biological aging [54], improved gut health [55], and potential benefits for cognitive [56], metabolic, cardiovascular [57], and certain cancer-related outcomes [58] across diverse populations not limited to Asian populations. Herbs and spices such as ginger, turmeric, and basil, widely used in Asian cooking, have demonstrated anti-inflammatory and immune-enhancing properties in RCTs [59,60]. Whole grains such as brown rice, a higher-quality source of carbohydrates, provide more fiber and micronutrients than refined grains such as white rice. A systematic review reported that higher brown rice intake was associated with a lower risk of T2D [61]. In coastal and riverine areas, fresh fish and aquatic products are important components of local diets, offering high-quality proteins, essential *ω*–3 fatty acids, vitamins, and minerals, all of which are important for overall health and healthy aging. Taken together, the health benefits of several traditional Asian foods have been suggested, although more high-quality studies are needed to confirm and evaluate these effects across diverse dietary patterns and traditional foods.

Beyond dietary patterns and foods, cooking methods are equally important determinants of diet quality. In many Asian traditions, cooking aims to preserve nutrients. Domestic cooking techniques, particularly in China and Korea, including steaming, boiling, low-temperature stir-frying [40], and stewing [62], help retain nutrients. Some water-soluble nutrients may be retained by steaming. Although boiling may lead to nutrient loss, stewing or boiling-based soup dishes may retain leached nutrients. An RCT showed that, compared with high-temperature cooking methods, a diet primarily based on steaming improved indicators related to T2D and CVD, including increased insulin sensitivity and elevated plasma levels of *ω*–3 fatty acids, vitamin C and vitamin E, while decreasing plasma cholesterol and triglyceride concentrations [63]. These traditional cooking methods embody the concept of “minimalism in preparation, maximization in health,” which contrasts sharply with the preparation methods of ultraprocessed foods.

Despite the health benefits of traditional Asian diets, they still face many challenges in gaining wider acceptance and promotion. Most current research is observational and focuses primarily on East Asia, whereas systematic studies on dietary habits and their impact on health in South and Central Asia remain limited. There is an urgent need to conduct high-quality interventional and observational studies in the future to comprehensively assess the impact of dietary patterns on health in different regions of Asia. The high heterogeneity across Asian regions in terms of ingredient selection, cooking methods, and cultural background makes constructing a unified dietary framework challenging. Currently, concepts such as the “Asian Diet Quality Score” or a culturally adapted Asian food pyramid are still in the exploratory stage. Therefore, dietary recommendations for Asia should adopt a culture-specific strategy, which is more effective than directly applying dietary guidelines, dietary pattern indices, or cooking methods from other regions.

In the 21st century, traditional Asian dietary patterns, including their food composition and cooking methods, have laid an important foundation for promoting health. We should build upon this foundation to systematically optimize and integrate diets, strategically incorporating evidence-based healthy elements from other cultures while retaining traditional healthy elements, thereby constructing a scientifically sound dietary pattern suitable for local populations.

## Concept of the AMED

As illustrated in Figure 1, the AMED diet is conceptualized as a dietary pattern that utilizes regionally available foods and traditional culinary practices while adhering to the principles of the Mediterranean diet [13]. Rather than prescribing specific Mediterranean foods, AMED translates these principles into the Asian context, with an emphasis on plant-based, minimally processed foods and healthier sources of carbohydrates, proteins, and fats. Table 1 compares the key components of traditional Asian diets and the Mediterranean diet, demonstrating how AMED can be operationalized within the Asian culinary context. Figure 2 presents the proposed AMED pyramid, highlighting the relative emphasis of its major food groups.

**FIGURE 1:**
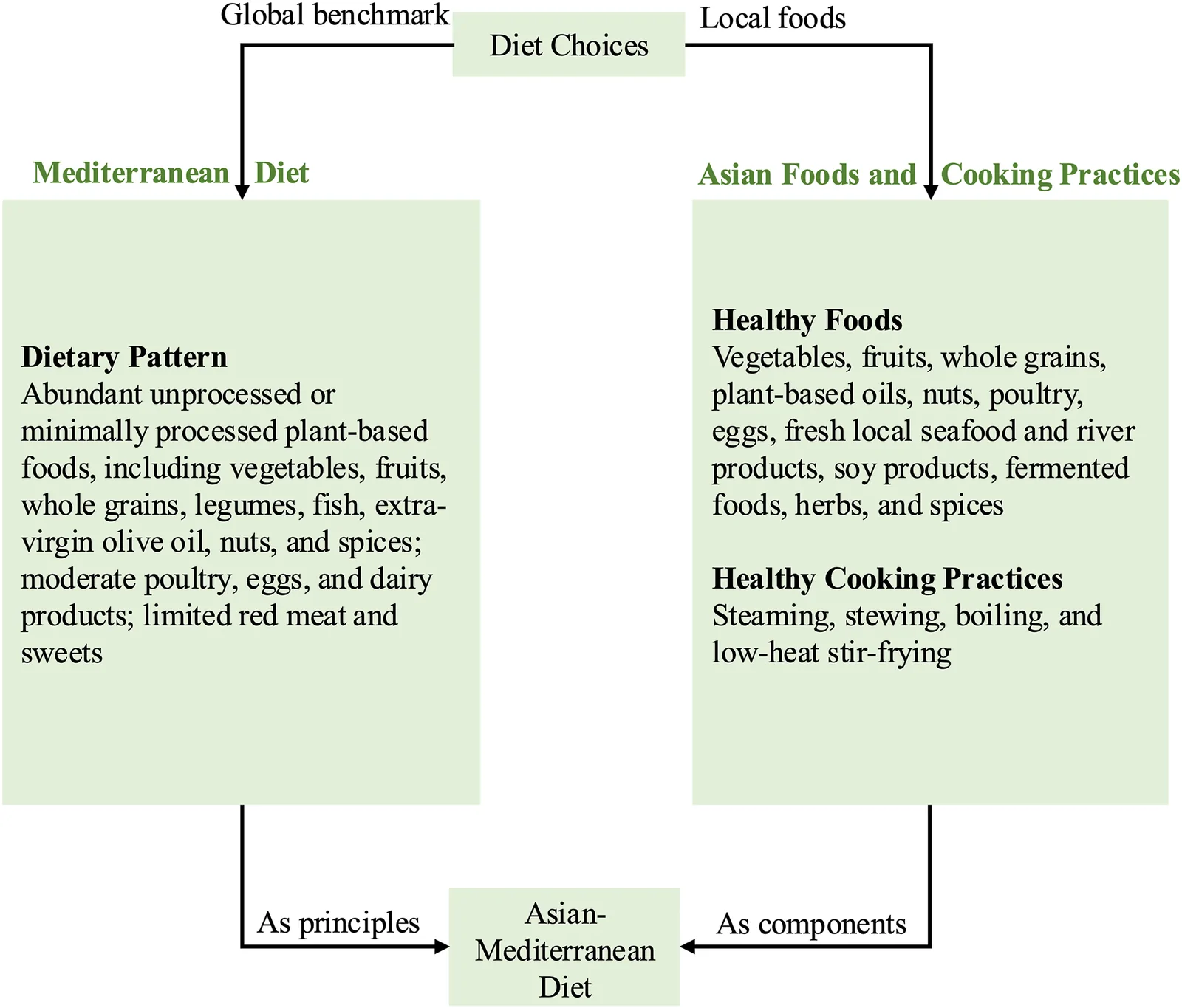
Conceptual framework of the Asian–Mediterranean diet. The AMED framework integrates the core principles of the Mediterranean diet with healthy, culturally familiar Asian foods and culinary practices. The Mediterranean diet provides the guiding dietary principles, whereas healthy regional Asian foods and cooking methods provide culturally appropriate components for local adaptation across Asia. AMED, Asian–Mediterranean diet.

**TABLE 1:** Comparison of traditional Asian, Mediterranean, and Asian–Mediterranean dietary patterns

Category	Traditional Asian diet	Mediterranean diet	AMED
Animal proteins	Marine products, river fish, shellfish, limited red meat	Fish, shellfish, moderate poultry, limited red meat	Fresh fish, river fish, shellfish, poultry, limited red meat
Plant-based proteins	Soy products (tofu, tempeh, natto), legumes (mung beans)	Legumes (lentils, beans, chickpeas), nuts (peanuts, walnuts, cashews)	Soy products (tofu, tempeh, natto), legumes (mung beans, chickpeas, lentils, beans), nuts (peanuts, walnuts, cashews)
Plant-based fats	Canola, sesame, peanut, rice bran, sunflower, and rapeseed oils	Olive oil, particularly extra-virgin olive oil, nuts	Canola, sesame, peanut, rice bran, and sunflower oils; olive oil, particularly extra-virgin olive oil; nuts
Carbohydrates	White rice, purple or brown rice, millet, buckwheat noodles, sweet potatoes	Whole wheat, barley, oats, brown rice, and bulgur	Brown or purple rice, millet, buckwheat noodles, sweet potatoes
Fruits and vegetables	Asian seasonal and tropical fruits, diverse vegetables	Seasonal fruits (grapes, figs, citrus), diverse vegetables	Asian seasonal and tropical fruits, diverse local vegetables
Bioactive-rich foods	Fermented foods (kimchi, natto, miso, and tempeh), teas (green, oolong, and pu-erh), herbs and spices (ginger, turmeric, and basil)	Herbs (oregano, rosemary, thyme), moderate wine	Fermented foods, teas, herbs, and spices
Dairy and alternatives	Soy milk, fermented soy products	Yogurt, cheese	Yogurt, soy milk, fermented soy products
Cooking methods	Steaming, boiling, stir-frying at low heat, stews, and soups	Grilling, roasting, sautéing with olive oil	Steaming, boiling, stir-frying at low heat, grilling, stews, and soups

Abbreviation: AMED, Asian–Mediterranean diet.

**FIGURE 2:**
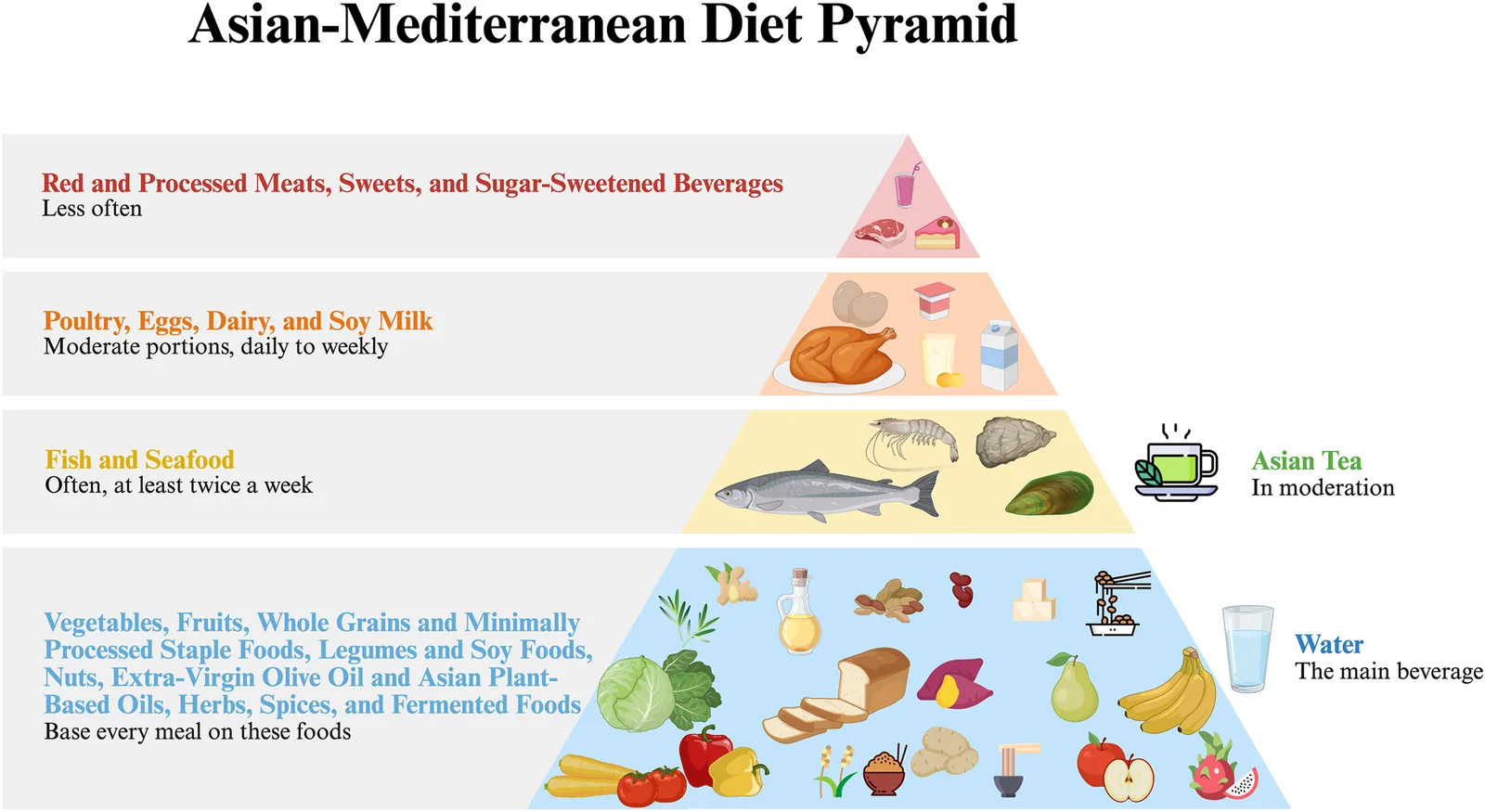
Asian–Mediterranean diet pyramid. The Asian–Mediterranean diet pyramid illustrates the recommended relative emphasis of the major food groups and beverages within the proposed dietary pattern, with examples of culturally familiar Asian food choices. The pyramid was inspired by the Mediterranean diet pyramid and adapted to reflect healthy Asian foods and culinary practices. Created in BioRender. LING, C. (2026). https://BioRender.com/qivym85

EVOO is a key component of the Mediterranean diet, with health benefits attributed to its high content in oleic acid and bioactive compounds like phenols. There are alternative plant-based healthy oils widely used in Asia with a long history of culinary application. These include canola, sesame, peanut, rice bran, and sunflower oils [40]. Although their fatty acid profiles vary, these oils share a high unsaturated-to-saturated fat ratio and are compatible with common Asian cooking techniques. An 8-wk parallel RCT conducted in Singapore compared the consumption of 30 g/d of blended vegetable oils made with refined rice bran, flaxseed, and sesame oils to 30 g/d of refined olive oil as the control; the study demonstrated that these oil blends can improve blood lipids and glucose profiles and lower body weight to a similar extent as the refined olive oil [64].

Similarly, peanuts and tree nuts such as walnuts and cashews, and seeds such as sunflower seeds, which are rich in MUFA and PUFA, are traditional and popular across East and Southeast Asia, both as snacks and culinary ingredients [65]. For plant-based proteins, traditional soy foods like tofu and tempeh, along with legumes such as mung beans and chickpeas, are widely consumed in the region and additionally provide fiber and essential micronutrients. Fresh seafood, including fresh fish, shellfish, and small river fish, is integrated into many Asian diets [40,66]. Together with poultry-based proteins popular among Asian cultures, these animal proteins align well with the Mediterranean diet recommendations on prioritizing poultry over red meat. Whole-grain staples, such as sweet potatoes, millet, brown or purple rice, and buckwheat noodles (soba), are affordable and widely prevalent across different Asian diets. These whole-grain foods are naturally low in glycemic index and provide satiety, making them suitable, local candidates for whole grains as recommended in the Mediterranean diet. Asia’s agricultural diversity supports a wide range of affordable seasonal fruits and vegetables, many of which are already embedded in local diets and are rich in fiber, antioxidants, and essential nutrients. These features support easy incorporation into the AMED framework. In addition, some traditional foods and beverages unique to Asian cultures, such as fermented foods and various teas (green, oolong, and pu-erh), are rich in polyphenols, probiotics, and other bioactive compounds [67,68]. These components have been linked to improved gut health, immune regulation, anti-inflammation, and cardiometabolic function, complementing the protective mechanisms established for the Mediterranean diet [68].

In addition to the choice of foods, traditional Asian cooking methods, when combined with a moderate use of sauces, particularly by limiting high-sodium, high-sugar, high-fat, and heavily processed condiments, can help support the diet’s overall nutritional quality. Traditional Asian herbs and spices rich in antioxidants and bioactive compounds can be introduced to enhance the flavor and further boost the health benefits of the AMED diet. This aligns with the Mediterranean dietary tradition, where aromatic herbs burst with flavor, such as oregano, thyme, and rosemary, serve as natural enhancers that reduce reliance on salt while amplifying taste and health benefits.

The integration of familiar ingredients, traditional cooking techniques, and local flavors is essential for ensuring AMED’s palatability, acceptability, and long-term sustainability [14]. Importantly, AMED is not proposed as a replacement for traditional Asian diets but rather as an evidence-informed adaptation of the Mediterranean diet, tailored for Asian contexts. It offers a flexible framework that can be easily adapted to various Asian settings and combines the strengths of both Mediterranean and Asian dietary patterns. Given the established evidence supporting the Mediterranean diet and overall health, AMED holds strong promise for promoting healthy aging and reducing NCD burdens in Asia upon future investigation.

## Challenges and Practical Considerations for Implementing the AMED

The AMED framework is designed to preserve the health-promoting features of traditional Asian diets and, equally important, to reduce those culinary practices contributing to NCD burdens through healthier, locally acceptable substitutions. Table 2 summarizes the major implementation challenges and corresponding dietary adaptations discussed in this section.

**TABLE 2:** Major implementation challenges and practical dietary adaptations for the AMED across diverse Asian settings

Implementation challenge	Common dietary practices	AMED adaptation
Refined carbohydrates	Refined wheat and rice flours, tapioca and potato starches used in traditional dishes and processed foods	Increase whole grains (brown/purple rice, barley, millet, and buckwheat) and minimally processed starchy foods such as sweet potatoes and yams
Sodium	Frequent use of salt, soy sauce, MSG, sodium-rich fermented foods and condiments, and salt-preserved meats and fish	Reduce discretionary sodium and salt-preserved meats and fish; retain fermented foods in moderation; enhance flavor with herbs, spices, aromatics, and other natural flavorings
Added sugars	Sugar added during cooking; traditional and Western-style desserts; sugar-sweetened beverages	Reduce added sugar; limit sugar-rich desserts and sugar-sweetened beverages; choose fresh fruit or nuts for dessert and water as the primary beverage
Cooking fats	Lard, ghee, and palm oil	Replace with locally available unsaturated plant oils such as rice bran oil or olive oil when feasible
Cooking practices	Deep frying; grilling over open flames; very high-temperature cooking; habitual consumption of very hot foods and beverages	Limit deep frying; prefer healthier cooking methods like steaming or stewing; avoid charring meat and fish; avoid very hot foods, beverages, and soups

Abbreviations*:* AMED, Asian–Mediterranean diet; MSG, monosodium glutamate.

One major challenge is the widespread habit of consuming large amounts of refined carbohydrates across diverse Asian populations. Refined wheat flour, refined rice flour, potato starch, tapioca starch, and other highly refined starches are widely incorporated into breads, noodles, traditional dishes, and processed foods across the region. Higher intakes of low-quality carbohydrates have been associated with adverse cardiometabolic risk factors and an increased risk of metabolic disorders, including among Asian populations [69]. Implementing the AMED framework may require gradual replacement of refined carbohydrates with regionally familiar whole grains and minimally processed starchy foods, whereas partial substitution within familiar dishes may offer a practical transition when full substitution is not yet feasible.

In addition, high sodium intake is common across parts of East and Southeast Asia because of the heavy use of sauces, fermented and preserved foods, and sodium-rich condiments. For instance, salt intake in South Asia significantly exceeds the WHO-recommended limit of 5.0 g/d [70], with intakes ranging from 6.7 to 13.3 g/d estimated in a 2023 review [71]. Excess sodium intake is linked to cardiovascular [72], metabolic [73], and immune dysfunction [74]. Reducing sodium intake will require gradual changes in both culinary practices and consumer taste preferences. Importantly, sodium-containing foods are not nutritionally equivalent. Sodium-rich fermented foods and condiments, such as kimchi and miso, may provide health benefits independent of their sodium content through bioactive compounds and probiotics, whereas salt-preserved meats and fish primarily contribute excess sodium and are generally associated with adverse cardiometabolic outcomes [72]. The AMED framework encourages moderation in the use of sodium-rich seasonings, while limiting salted foods and retaining culturally important fermented foods in appropriate portions. Flavor can instead be enhanced with herbs, spices, aromatics, vinegar, citrus, and naturally umami-rich ingredients such as tomatoes, mushrooms, and seaweed, thereby reducing reliance on added salt while preserving palatability.

Added sugars are commonly consumed across many parts of Asia, including in sauces and during food preparation. Traditional and Western-style desserts, together with increasing consumption of sugar-sweetened beverages, further contribute to added sugar intake. Higher added sugar intake has been associated with obesity, cardiometabolic disorders, impaired cognitive function, and frailty [75,76]. The AMED framework therefore advocates reducing added sugars in cooking where feasible, limiting sugar-rich desserts and sugar-sweetened beverages, and promoting healthier snacks such as nuts and fresh fruits, with water as the primary beverage. However, in some parts of Asia, limited access to safe drinking water remains an important implementation barrier that may influence beverage choices, highlighting the need for broader public health improvements beyond dietary recommendations.

Finally, dietary quality also depends on culinary practices. Deep frying should be limited in favor of healthier cooking methods, such as steaming, boiling, and stewing [77]. Cooking meat or fish at very high temperatures or over an open flame may increase the formation of potentially carcinogenic compounds, including heterocyclic amines and polycyclic aromatic hydrocarbons [78,79]. Likewise, cooking fats high in SFAs, such as lard, ghee, and palm oil, which are commonly used across parts of Asia, should be limited and replaced with locally available oils rich in unsaturated fatty acids or olive oil, recognizing that availability and affordability may influence these choices. Habitual consumption of very hot beverages and soups should be avoided, as it has been associated with an increased risk of esophageal injury and esophageal cancer [80].

To derive evidence-based data on the health effects of AMED, we have recently launched a 20-wk, 2-arm RCT evaluating a locally adapted AMED in Singapore. The intervention group receives prepared meals designed under the AMED framework, incorporating local ingredients and culinary preferences while emphasizing whole grains, vegetables, plant-based proteins, and key Mediterranean components, including EVOO and nuts. The control group receives standard dietary advice following national guidelines. Follow-up visits collect anthropometric data, health and lifestyle measures, and biospecimens. Primary outcomes include changes in adiposity and glycemic and cardiometabolic biomarkers, while secondary outcomes include gut microbiota and brain health biomarkers. AMED adherence is assessed using follow-up questionnaires, meal compliance, and biomarker analysis. The trial is expected to conclude by the end of 2026, with findings expected to inform future AMED implementation across Asia and support the development of large, multicenter, community-level interventions.

In conclusion, in the face of rising NCDs and population aging and given the central role of nutrition in disease prevention and health promotion, accessible, affordable, locally adapted, and culturally sustainable dietary strategies are urgently warranted to promote health and longevity in Asia. The AMED diet presents a promising approach, bridging the strengths and unique features of traditional Asian diets with the evidence-based health benefits of the Mediterranean diet. By preserving local culinary heritage while integrating scientifically supported healthy components from the Mediterranean diet, the AMED framework offers a culturally relevant, flexible model that supports the development of healthy eating patterns across diverse Asian populations. Leveraging regional food systems, the AMED framework not only promotes individual and public health but also supports local agriculture and aligns with the broader goals of dietary sustainability and planetary health [81].

Further studies are needed to evaluate AMED’s health effects, feasibility, and cultural acceptability and sustainability. Policymakers and government agencies play a central role in supporting healthy diet adoption at scale. Supportive policies may include funding for research, incorporating AMED concepts into national dietary guidelines, launching public education and media campaigns, and improving access to healthy foods and beverages across all socioeconomic groups. To align public health nutrition with environmental sustainability, food system monitoring and support for local agriculture and fisheries are also essential.

The AMED framework holds potential benefits that not only improve health across the lifespan but also extend beyond individual health, supporting intergenerational well-being and strengthening community food systems. With coordinated action, AMED has the potential to serve as a viable and scalable solution to improve dietary quality and foster longer and healthier lives in Asia and globally.

## Author contributions

The authors’ responsibilities were as follows – CZ: conceptualized and supervised the work and provided funding support for the work; JY, CL: drafted the manuscript; YTE, YSC, FBH, LH, MAM-G, CJH, CZ: provided input for critical revision of the manuscript; and all authors: contributed to the final content and read and approved the final version of the manuscript.

## Declaration of generative AI and AI-assisted technologies in the writing process

During the preparation of this work, the authors used ChatGPT (OpenAI, GPT-5) to assist with language editing and improve the clarity and readability of the manuscript. After using this tool, the authors reviewed and edited the content as needed and take full responsibility for the content of the publication.

## Funding

This work was supported by the Yong Loo Lin School of Medicine, National University of Singapore. The funder had no role in the design, conduct, or reporting of the study and had no influence over the decision to publish.

## Conflict of interest

The authors report no conflicts of interest.
